# Addressing Mood Disorder Diagnosis' Stigma With an Honest, Open, Proud (HOP)-Based Intervention: A Randomized Controlled Trial

**DOI:** 10.3389/fpsyt.2020.582180

**Published:** 2021-02-10

**Authors:** Arlete Modelli, Viviane P. Candal Setti, Martinus Theodorus van de Bilt, Wagner Farid Gattaz, Alexandre Andrade Loch, Wulf Rössler

**Affiliations:** ^1^Laboratory of Neuroscience (LIM 27), Institute of Psychiatry, University of São Paulo, São Paulo, Brazil; ^2^Psychology and Neuropsychology Service, Department and Institute of Psychiatry, Universidade de São Paulo Medical School, São Paulo, Brazil; ^3^Instituto Nacional de Biomarcadores em Neuropsiquiatria (INBION), Conselho Nacional de Desenvolvimento Cientifico e Tecnológico, São Paulo, Brazil; ^4^Department of Psychiatry, Psychotherapy and Psychosomatics, Psychiatric Hospital, University of Zurich, Zurich, Switzerland; ^5^Department of Psychiatry and Psychotherapy, Charité University of Medicine, Berlin, Germany

**Keywords:** self-stigma, disclosure, stigma stress, mental illness, self-conscience, Honest, Open, Proud

## Abstract

**Introduction:** The public stigma and self-stigma contribute to the dilemma of disclosing or not one's own mental illness diagnosis. Studies suggest that revealing it diminishes stress, besides helping with self-esteem. Honest, Open, Proud (HOP) is a group program that aids in the process of deciding on it, reducing its impact. Considering the relevance of this issue, the present study aimed to apply a HOP-based intervention in a group of patients diagnosed with mood disorders.

**Methods:** A randomized controlled clinical trial was used, including 61 patients with mood disorders, of whom 31 were diagnosed with depression and 30 were diagnosed with bipolar disorder. They were randomly placed on the intervention (HOP) or the control group (unstructured psychoeducation). The evaluations occurred before (T0) and after (T1) the sessions. We administered eight scales, from which three presented relevant results: Coming Out with Mental Illness Scale (COMIS), Cognitive Appraisal of Stigma as a Stressor (CogApp), and Authenticity Scale.

**Results:** The intervention groups (depression and bipolar) did not present a significant change regarding the decision to disclose their diagnostics. However, the depression group showed a decrease on the perception of stigma as a stressor (T0 = 0.50 vs. T1 = −1.45; *p* = 0.058). Improvements in post-intervention results were seen for both groups (depression and bipolar) on the Authenticity Scale—self-alienation subscale (T0 = 10.40 vs. T1 = 12.37, *p* = 0.058).

**Conclusion:** Our HOP-based intervention appeared to be an important program to aid patients in facing stigma stress, showing positive effects, whether helping to diminish stress or to improve self-conscience, both of which have indirect effects on self-stigma. As it is a compact program, it can bring benefits when applying to public health institutions.

## Introduction

Stigma is a multifaceted construct, a mark that assigns its bearer a condition of depreciation, compared with other members of society (1). In the stigma process, individuals are identified based on an undesirable characteristic of them and are labeled and discriminated, being unappreciated by society. This kind of stigma is called social or public stigma (2–7). An important remark is that the stigma incurs in a vicious cycle of prejudice and discrimination, reinforcing the occurrence of the stigma itself (8, 9).

As someone with a mental disorder becomes self-aware of the negative beliefs others might have toward mental illness, he/she avoids reaching out to health services, to support on work environment, to professional development and emotional relationships (10, 11). Also, possible increase of relapses and hospital admissions is expected (12). The occurrence of such processes is often connected to the patient's agreement with these labels and demonstrates self-stigma—the loss of self-respect and self-rule, among other things (13–17). The consequences are harmful, affecting different aspects of someone's life, besides creating a dilemma about disclosing or not their diagnosis.

Studies suggest that concealing a mental disorder diagnosis as a way to avoid the stigma tends to increase the stress associated with cognitive, emotional, and behavioral aspects, as well as negative self-evaluation (18). Pachankis (19) emphasizes the consequences of occult stigma and the dilemma of disclosing stigmatizing aspects at relevant moments, such as work–life, relationships, and school, and ascertains that cognitive difficulties (decision-making) can lead to affective and self-evaluating distress.

Specific studies on patients diagnosed with mood disorder point out that stigma is an important issue in this population, either as public stigma or self-stigma (14, 15, 20, 21). However, few of those address interventions that can help cope with this situation.

Although there is an increase on possible interventions that contribute to the discussion on revealing one's mental disorder, along with actions that assist on dealing with this decision, a study focusing on the label of mood disorders appears to be necessary.

An important tool in this sense has been the Coming Out Proud intervention. It consists in a brief group intervention (three sessions), designed to diminish the stress related to the dilemma of disclosing or not self-diagnosis of mental illness. A previous version of the program was developed by Corrigan and Lundin, based on a book (2001) and named Coming Out Proud (COP). However, a more updated version was submitted and called Honest Open Proud (HOP)^1^. Studies that utilize the HOP Program (22–24) indicated a decrease of stress due to prejudice (stigma stress), mainly when referring to possibly disclosing a mental illness, besides pointing out tendencies on improving levels of self-stigma, as well as self-rule and independence. There are few studies based on the application of this program, especially considering groups of patients with the same diagnosis (25).

Therefore, this study aimed to identify on a group of patients diagnosed with mood disorders (depression and bipolar) whether HOP-based interventions would allow greater flexibility to socially expose or not one's diagnosis, and whether the interventions would reduce the stress related to secrecy and improvements to self-rule.

## Method

### Study Design and Sample

Patients diagnosed with unipolar depression and bipolar disorder according to the Diagnostic and Statistical Manual of Mental Disorders (DSM-V) (26) participated in this randomized controlled clinical trial.

Inclusion criteria consisted of the following: minimum age of 18 years old; capacity to provide informed written consent; currently undergoing outpatient follow-up; currently being euthymic; presenting at least a moderate level (grade 4) on a screening question: “How concerned or stressed have you normally felt when deciding to tell others about your mental illness or to keep it a secret?” (1 = not stressed or concerned; 7 = very stressed or concerned).

The exclusion criteria included the following: intellectual deficit, current presence of mood symptoms, and comorbid alcohol or drug use related disorders. Information regarding inclusion and exclusion criteria were gathered through interview and accessing the patient's hospital file.

The subjects were recruited from mid-2018 to the end of 2019, at the Institute of Psychiatry of the University of Sao Psaulo. They were selected through a research call that was broadcasted within the institutional environment and via search through the institution's patients list. Nine patients with depression and three with bipolar disorder responded the research call. Regarding the list search, 90 bipolar patients and 70 with depression were contacted by telephone. Overall, 51 individuals diagnosed with depression and 57 with bipolar disorder corresponded to the inclusion and exclusion criteria, responded to the triage question a grade equal or superior to 4, and were put on two randomized diagnosis-specific lists—one with bipolar individuals and the other with depression individuals.

### Randomization

Randomization was provided by the Clinical Trial Randomized Service^2^, which randomly assigned numbers to two lists (intervention or control). Each participant in each diagnostic group was consecutively given a study number, according to their entry in the study and agreement to participate, and assigned to the control or intervention listing accordingly. Each time a group of six to eight patients was filled in, in either the intervention or control, individuals were called upon to start the study.

**Figure 1:**
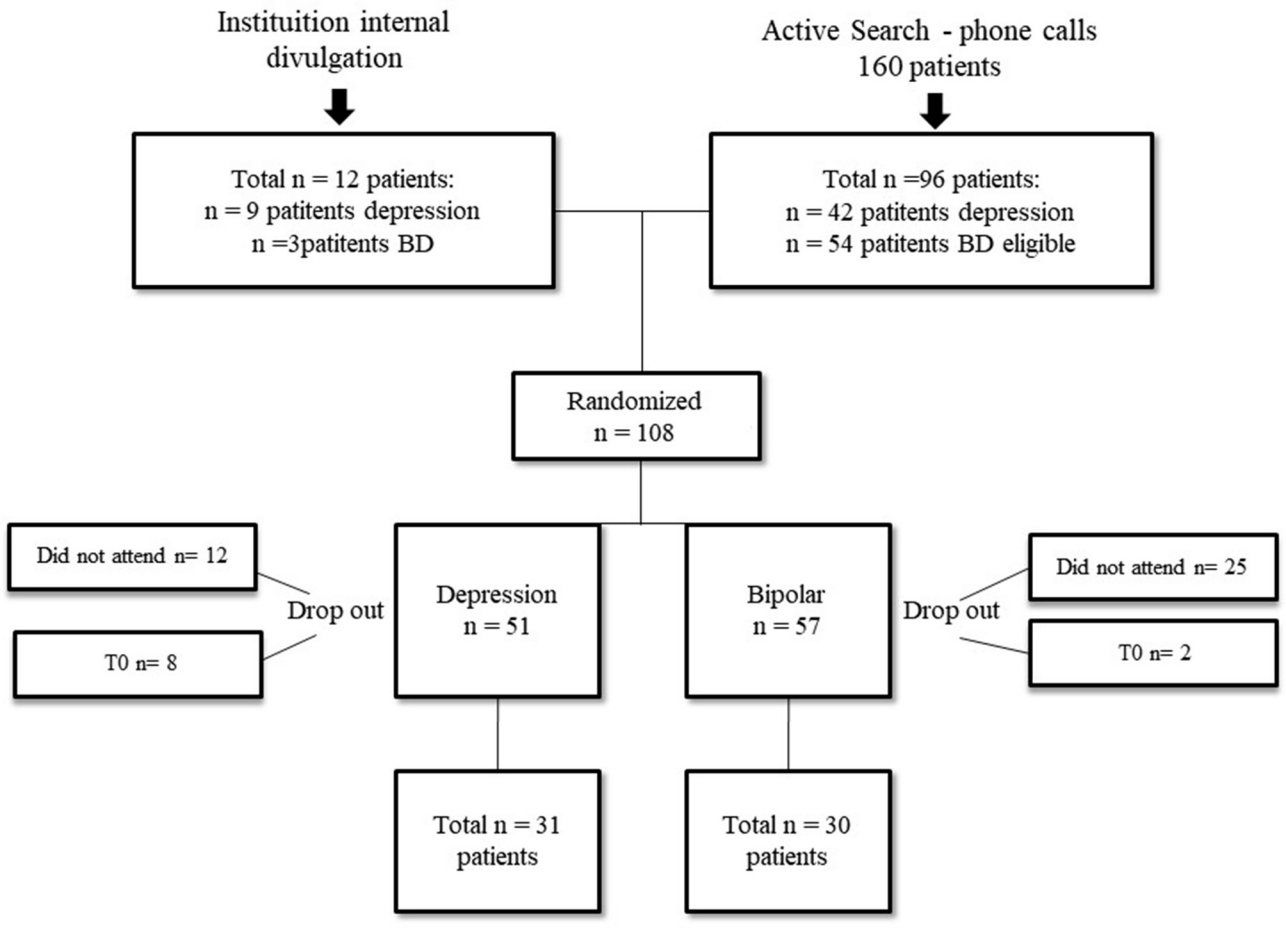
Patient flowchart.

Throughout this process, 12 depression patients and 25 with bipolar disorder withdrew participation (dropouts). After the groups began, eight depression individuals and two bipolar patients attended only the first meeting (dropouts). Accordingly, at the end, our sample consisted of 31 participants on the control group (16 with depression and 15 with bipolar disorder diagnosis) and 30 on the intervention group (15 with depression and 15 with bipolar disorder) (Figure 1).

The intervention group of 30 patients underwent a program based on the HOP. The goal of this intervention is to train the patient to be able to lead the group. However, the main researcher (A.M.) was the intervention leader. This procedure is distinct from all previous studies until now (22–24, 27), as the leadership coming from someone with a mental disorder diagnosis can facilitate the bonding process, specially through identification.

However, the decision of appointing a mental health professional was made to allow a better discrimination and understanding of their beliefs and distresses; to review aspects related to biases against mental health and mental health professionals (6, 28); and to allow programs such as HOP, which proposes a short intervention, to be utilized on public health institutions and facilitate the proximity between multi-disciplinary team and patients—for the training of a patient as a facilitator could significantly hamper and delay the process in these settings.

HOP has the main objective of supporting people with mental disorders on deciding to disclose or not their mental illness and treatment. The intervention consisted on a 2-h session on a weekly basis for a 3-week period. The groups consisted of six to eight individuals, and all the participants received a copy of the HOP work folder. Each lesson, according to the manual, dealt with specific topics, such as (1) risks and benefits of disclosing or keeping a secret about their diagnostics on different situations; (2) levels of disclosure, on a scale from complete social withdraw to indiscriminated report of their experience with mental illness; and (3) useful ways of telling their story about mental illness in different scenarios.

For the control group, the same number of sessions and workload was applied, but they were used to make an unstructured discussion on subjects such as mental illness, treatment, adherence to treatment, and family relationship.

### Instruments

All the instruments were applied at two moments: T0 = initial evaluation, 3 days before the first group session; T1 = post-intervention evaluation, within a 3-day period after the third session.

The evaluations were personally applied to ensure the data were complete and the participants could ask questions, in case of doubts. Eight scales were administered to measure different individual aspects. The scales were translated from English to Portuguese, then translated back to English and verified regarding their consistency by a bilingual psychologist. After the necessary adjustments, a pre-test of the scales was performed on a random population (three hospital employees and three students where the research was developed). Therefore, we verified the instrument's (1) application period, (2) viability, and (3) language adequacy to the studied population.

The following instruments were applied: (a) Subjective Quality of Life−17 items (29), examining the frequency of social contact, satisfaction with social relationships, amount of leisure activities and respective level of fulfillment; (b) Rosenberg's Self-Esteem Scale (30, 31), which evaluates how someone feels about themselves through 10 sentences, rated from 1 (completely disagree) to 4 (completely agree); (c) Coming Out with Mental Illness Scale (COMIS) (18), measuring the perceived benefits of coming out, followed by 42 declarations regarding the motives (1 = completely disagree, 7 = completely agree); (d) Authenticity Scale (32), a 12-sentence assessment of individual authenticity concerning relationship problems and coping with them (1 = completely disagree, 5 = completely agree); (e) Self-Stigma of Mental Illness Scale—Short Form (SSMIS) (33), which evaluated if the participants applied negative stereotypes, through 20 affirmations, each of them being graded 1 = completely disagree and 9 = completely agree; (f) Internalized Stigma in Mental Illness Scale−29 items (ISMIS) (34–36), measuring the individual's internalized stigma through 29 sentences, 1 = completely disagree and 5 = completely agree; (g) Stigma Stress Scale (CogApp) (37), an eight-item scale, each one rated from 1 to 7, examining the cognitive evaluation of the stigma as a stressor; and (h) Barriers to Access Care Evaluation (BACE) (38, 39), 30 items that inquire about the decision of looking for professional help and possible related difficulties, with scores of 0 = no difficulties to 3 = great difficulty.

Social–demographic data were also gathered, such as age, gender, marital status, years of study, and social–economic status. This last was classified according to the Brazilian Institute of Geography and Statistic (IBGE)^3^, where class A = higher income, and E = lowest income.

This study was approved by the Research Ethics Committee of the Hospital das Clínicas, from the University of São Paulo Medical School (CAPPesq HC FMUSP; CAAE n° 57068216.3.0000.0068).

### Statistical Analysis

Data were described in terms of mean and standard deviation, for continuous variables, and number and percentage for categorical variables. Differences across groups (intervention vs. controls) were analyzed with ANOVA and Student's *t*-test, and with chi-square for continuous and categorical variables, respectively. Regarding pre- and post-trial comparison, differences between T1 and T0 scores were calculated for controls and for the intervention group, for each individual. As this difference was not normally distributed, Student's *t*-test was used for statistical comparison. All analyses were performed with SPSS version 23 for Mac.

## Results

The demographic data are summarized in Table 1. Overall, no statistical differences were seen between groups in sociodemographics. It shows a similar predominance of women in both groups, and similar mean ages (42.2 vs. 42.8). The intervention group showed more individuals with higher education, 80% (24 patients) vs. 58% (18 patients) of the control group. The majority of the intervention group was single (56.7%), unlike controls (38.7%). Also, the intervention group showed that 14 patients (46.7%) were currently employed and, in the control group, that statistic corresponded to 12 patients (38.7%). However, these differences were not statistically significant.

**Table 1:** Sample characteristics.

	**Intervention**	**Control**	***P***
Sex (female; *n*, %)	21 (70%)	24 (77.4%)	0.51
Age (mean, SD)	42.2 (16.8)	42.8 (11.9)	0.87
Years of education (13 or more; *n*, %)	24 (80%)	18 (58%)	0.21
Marital status (single; *n*, %)	17 (56.7%)	12 (38.7%)	0.51
Diagnosis (bipolar; *n*, %)	15 (50%)	15 (48.4%)	0.90
Employed (yes; *n*, %)	14 (46.7%)	12 (38.7%)	0.53

Tables 2, 3 show data of the Cognitive Appraisal of Stigma scale (CoGapp). This scale accesses the stress experienced from prejudice against mental health disorders (HARM— “Prejudice against people with mental disorders will have harmful consequences to me”), as well as the possibility to demonstrate abilities to coping with it (COPING— “I have the resources needed to deal with problems caused by prejudice against people with mental illness”). Stigma-related harm showed a greater decrease in the intervention group compared to the control group (4.68–3.58 vs. 4.56–4.40, respectively). In coping resources, we also observed an increase, which was greater in the intervention group (4.18–5.03 vs. 4.01–4.36, respectively). Difference was marginally significant (*p* = 0.058).

**Table 2:** Scores on the Cognitive Appraisal of Stigma scale on individuals with depression (COGAP).

**COGAP**	**Intervention**		**Control**	
**(mean, SD)**	**T0**	**T1**	**T0**	**T1**
Stigma-related harm	4.68 (2.11)	3.58 (1.65)	4.56 (2.14)	4.40 (1.79)
Coping resources	4.18 (1.27)	5.03 (1.41)	4.01 (1.65)	4.36 (1.47)
Stigma stress (=harm-coping)	0.50 (2.40)	−1.45 (2.38)	0.55 (3.36)	0.05 (3.03)

*SD, standard deviation.

**Table 3:** Student's *t*-test for differences in the COGAP on individuals with depression.

**COGAP (mean, SD)**	**Intervention**	**Control**	***P***
Stigma-related harm	−1.10 (1.59)	−0.15 (1.34)	0.084
Coping resources	0.85 (1.16)	0.34 (1.20)	0.243
Stigma stress	−1.95 (1.86)	−0.50 (2.21)	0.058

*SD, standard deviation.

On BD individuals, there was no significant difference between intervention and control regarding the Cognitive Appraisal of Stigma. However, baseline scores for BD were better than those of depression individuals. Stigma-related harm was lower in BD individuals compared to those with depression (4.62 vs. 4.12, respectively, *p* = 0.35) and Coping resources was significantly higher for BD individuals than for individuals with depression (4.95 vs. 4.10, respectively, *p* = 0.02).

As for the decision of disclosing the diagnosis itself, at baseline most of the sample had previously decided to reveal their diagnosis (40 subjects, 66%). After the HOP intervention and the control group, only four new individuals changed their idea and decided to reveal their diagnosis (two of them from the intervention group and two controls). As such, difference was not statistically different (*p* > 0.05).

Tables 4, 5 show the results for the Authenticity Scale, whose goal is to measure a tripartite conception of authenticity: self-alienation, authentic living, and accepting external influences. For the whole sample, we observed that, among those three aspects, self-alienation (self-conscience) demonstrated an improvement after the intervention, which had marginal statistical difference (*p* = 0.058).

**Table 4:** Scores on the Authenticity sub-scales (whole sample; higher scores indicate less stigma).

**Authenticity**	**Intervention**		**Control**	
**(mean, SD)**	**T0**	**T1**	**T0**	**T1**
Authentic life	16.20 (2.48)	15.80 (2.80)	16.90 (3.28)	15.61 (3.50)
External influence	13.00 (3.76)	13.80 (4.09)	14.64 (3.82)	14.96 (3.42)
Self-alienation	10.40 (4.12)	12.37 (4.47)	12.80 (4.62)	13.32 (4.11)

*SD, standard deviation.

**Table 5:** Student's *t*-test for differences in the Authenticity scales (whole sample).

**Authenticity (mean, SD)**	**Intervention**	**Control**	***P***
Authentic life	−0.40 (2.93)	−0.29 (2.86)	0.883
External Influence	0.80 (2.72)	0.32 (2.38)	0.469
Self-alienation	1.97 (3.37)	0.51 (2.40)	0.058

*SD, standard deviation.

All other results from the different instruments showed no statistical difference between intervention and control group.

## Discussion

To the best of our knowledge, this study was the first to use a HOP-based program on patients with the same diagnostics, namely, mood disorders. It is worth mentioning that studies on patients diagnosed with the same disorder allow us to recognize specific details, identifying specific interventions and approaches, if necessary (27).

Results showed that our HOP-based intervention improved stigma stress in individuals with depression and improved self-alienation in both BD and depression individuals. Both results had marginal statistical significance. HOP did not significantly interfere with the decision to disclose or not the diagnosis, though. During the conduction of the program, patients from both groups broadly uncovered that living with stigma causes suffering. They discussed experiences of prejudice and discrimination, lived among family members and social situations. Besides provoking discomfort, they also showed the patient's self-stigma, who agreed with beliefs of laziness and lack of interest (depression), or unruly, uncompromised or incapable behavior (bipolar). On that note, the discussion regarding recognizing themselves with the illness, the beliefs, and the pros and cons on disclosure (HOP lessons) pointed out the patient's self-stigma (27, 40), which puts on debate the deconstruction of pre-constructed imagery.

Regarding the decrease of stigma stress on the depression group, this suggests improvements to coping mechanisms on prejudice and discrimination experiences. Other studies addressing diagnosis' disclosure highlight aspects of self-stigma and self-competence enrolled in this process (22–24, 27). However, in the present work, the number of patients that decided to reveal the diagnosis after the intervention did not significantly increased. This might have happened because, at baseline, 66% had already disclosed their diagnosis. Nonetheless, despite this disclosure, the stress of dealing with others' reactions could still be a relevant issue. The intervention could thus help develop skills and indicate a few ways to deal with these situations, perhaps aiding on feelings of guilt, very common to depression.

Another aspect that may be present in these results suggests, as a hypothesis, the presence in the depression group of cognitive distortions (psychological suffering) that are particularly important that could influence a worse perception on the attitudes of others, showing themselves to be more sensitive to other's behavior. This observation is mentioned by Major and O'Brien (41) and Rusch et al. (37) when describing some points related to the understanding of stress with stigma, as well as one of the results indicated by Griffiths et al. (42). Rüsch et al. (37) also mentioned that among the emotional reactions to the stress of the stigma, shame is pointed out when the perception of stigma is seen as more harmful. It is possible to hypothesize that the depression group tends to misinterpret the trivial, neutral, or even the more stressful daily life events at first, usually as evidence of personal effects, demonstrating an exaggerated sense of responsibility for adversity, afterwards being “improved,” from the moment that beliefs and concepts can be addressed in targeted activities.

Mendoza-Denton et al. (43) studied another aspect and presents, in an article on status-based sensitivity to rejection, the presence of expectations about rejection based on personal characteristics, as well as based on direct or indirect experiences related to status characteristics, in which the expectation of rejection would be linked to experiences in situations where there are no sharing of their stigma, but stories of exclusion or marginalization. These aspects seem to help in understanding, considering that the dynamics of patients with depression are linked to narratives of guilt, worthlessness, hopelessness, disinterest, and lack of value.

However, the bipolar intervention group did not present significant results on diminishing the stress on the dilemma of disclosing the diagnostics. For our BD patients, the disclosure was generally described as “something out of their control,” as friends, work/college colleagues and family members witnessed their symptoms—specifically the manic ones—during the illness' critical moments. They also mentioned feelings of embarrassment and shame. At the same time, telling others about the disorder occasionally granted more collaboration at the school/work environment (40, 44).

We emphasize that, although the indexes do not point to significant results, the bipolar group presented, during the study, lower results related to stress, considering less damage and better coping results, both in T0 and T1. These results, even not significative, would point to better cognitive resources demonstrating a “more elaborate” way to face stigma. Major and O'Brien (41) refer to a model where there would be possibilities for involuntary and voluntary responses. Would the Bipolar group have better resources for voluntary responses showing more coping skills compared to the depression group? The author states that voluntary responses would demonstrate conscious efforts with better control over emotions, cognition, and behavior.

Studies pointed out that people with occulted mental illness stigma—which Goffman (1) called “discredited” —, by keeping the condition a secret, would feel apprehensive that they can be discovered during social or work situations, fearing the consequences of this revelation. This reinforces that the “fear of being discovered” —or disclosure—is an independent stress factor to those with occult stigma, on which the condition of being stigmatized is not completely known on every social situation (as opposed to the visible stigma) (19). Therefore, we can hypothesize that the BD individuals presenting stigma stress is more related to the consequences of an episode—losses to finances, work, and relationships—as described by many patients (45), than to disclosure itself.

Still, patients from both groups informed feeling good about sharing experiences with others that suffer with similar symptoms, which provides a sense of belonging. Studies point out that the presence of other people that share the stigma tends to elevate self-esteem and bring out a more positive mood (46), favoring interactions with such group (47). Corroborating with the arguments above, Rüsh et al. (22) refer that people with an extensive record of mental disorders can benefit from HOP, due to having many experiences with stigma, disclosure and secrecy, and to being able to discuss them with a group, besides bringing up opportunities to relate with people with mental disorders.

Mulfinger et al. (24), after using HOP on teenagers with mental disorders, also highlighted positive results on lessening the stress toward the diagnostics and their decision on disclosing it, and affirmed its benefits at the start of the treatment. Adapted to this situation, HOP brought up important discussions, expanding the disclosing environment and considering social media as a valid instrument.

The Authenticity Scale, which measures how authentic someone is toward coping skills, recognition, and daily life responsibilities, reflected another important result. After our HOP-based intervention, improvements to self-alienation were observed.

According to Wood et al. (32), authenticity is not an attribute, but a process of continuous making, consisting of a tripartite conception including self-alienation, authentic living, and accepting external influence. We could assume that authenticity would be the balance of an authentic life (“I always maintain what I believe in”), along with the ability of not being influenced by external sources (“I am strongly swayed by other's opinions”) and the ability to not alienate themselves (“I don't know how I really feel inside”). Each of these aspects reveals a condition of dealing with stigma, apart from self-stigma.

The improvement of self-alienation indicates how important it can be to instigate discussions aimed to expand knowledge of the illness and one's relations to it, for instance, recognizing stereotypes that revolve around prejudice against mental disorders, the emotional reactions after disclosing the diagnostics, and behavioral intentions recognized on oneself and on others. This was enabled by group identity. Watson et al. (48) indicate this as relevant to coping with stigma and self-alienation, developing more positive self-perceptions. There were several accounts of self-experienced stigma, how they dealt with it and alternatives they were able to conceive after the HOP intervention. Corroborating with the group identity concept, Corrigan et al. (23) state that the group experience can enhance personal resilience toward stigma and self-stigma, especially due to having shared their stories.

There were some limitations to this study that must be considered. First, a considerate amount of time for active search was necessary. This denotes that there is not a habit of research participation in our country, but also that people are not willing to talk about stigma. As such, the people that are willing to engage to these projects are usually already involved in some way and want to reflect on it or change it, as opposed to those who believe there is nothing to be done. Non-participants could hypothetically have higher levels of self-stigma, as mentioned by Corrigan's term “why try?” (23, 49, 50).

Another limitation concerns the small number of the sample. We recognize that a greater number of subjects is necessary to allow a better interpretation of the results, as well as to possibly enhance the statistical reach of our findings.

An aspect observed by Rüsh et al. (22) and Mulfinger et al. (24) refers to the number of people that previously decided to disclose the diagnostics. Considering the aim of this program, it would be relevant that the intervention also included a number of people with doubts on this topic.

## Conclusion

Our study corroborated with findings that HOP can contribute to diminishing stress on the dilemma of disclosing or not the diagnosis of depression. Sharing narrative constructions regarding oneself with a group can also be beneficial and influence self-alienation, for both depression and BD patients. We would like to acknowledge that stigma toward mental illness is still an enduring issue worldwide and that the stress related to the disclosure of self-diagnosis depends on the level of public stigma, the perception, and introjection of it by the subject. On the one hand, HOP-based studies should be multiplied with larger samples and with different diagnoses to prove its efficacy and specificity, and mainly to avoid this introjection. On the other hand, public campaigns should be promoted to dispel the stigma toward mental illness.

## Data Availability Statement

The original contributions presented in the study are included in the article/supplementary materials, further inquiries can be directed to the corresponding author.

## Ethics Statement

The studies involving human participants were reviewed and approved by Research Ethics Committee of the Hospital das Clínicas, from the University of São Paulo Medical School (CAPPesq HC FMUSP; CAAE no 57068216.3.0000.0068). The patients/participants provided their written informed consent to participate in this study.

## Author Contributions

WR, AL, AM, VS, MB, and WG: study conception and design. AM, AL, and VS: acquisition of data. AM, AL, and WR: analysis and interpretation. All authors contributed to the article and approved the submitted version.

## Conflict of Interest

The authors declare that the research was conducted in the absence of any commercial or financial relationships that could be construed as a potential conflict of interest. The reviewer CR declared a shared affiliation, with no collaboration, with several of the authors AM, VS, MB, WG, and AL to the handling editor at the time of the review.

## Footnotes

^1^www.comingoutproudprogram.org

^2^https://www.randomize.net/

^3^www.ibge.gov.br
